# Association between carcinoembryonic antigen, carbohydrate antigen 19-9 and body mass index in colorectal cancer patients

**DOI:** 10.3892/mco.2013.158

**Published:** 2013-07-23

**Authors:** WEI CHEN, QIN LIU, SHU-YUN TAN, YAN-HUI JIANG

**Affiliations:** 1 Department of Colorectal Surgery, The Sixth Affiliated Hospital of Sun Yat-sen University, Guangzhou, Guangdong 510655, P.R. China; 2 Department of Pathophysiology, Zhongshan School of Medicine, Sun Yat-sen University, Guangzhou, Guangdong 510080, P.R. China; 3 Management School, Hunan University, Changsha, Hunan 410079, P.R. China

**Keywords:** colorectal cancer, carbohydrate antigen 19-9, carcinoembryonic antigen, body mass index

## Abstract

Carbohydrate antigen 19-9 (CA19-9) and carcinoembryonic antigen (CEA) have been well recognized as tumor markers for colorectal cancer. Previous studies suggested that obesity is inversely associated with the screening of CEA and CA19-9 levels and may reduce screening sensitivity. This study was conducted to evaluate the association of body mass index (BMI) with serum CEA and CA19-9 concentration in colorectal cancer patients. A total of 300 patients were enrolled in the study, selected from 2,950 consecutive colorectal cancer patients who underwent surgical treatment between August, 1994 and December, 2005. The association of BMI with CEA concentration, total circulating CEA mass and plasma volume was assessed by determining P-values for trends. The multivariate linear regression analysis was used to adjust for clinicopathological confounding factors to analyze the main outcome measures when CEA and CA19-9 had been log-transformed. Increased BMI was linearly correlated with a higher plasma volume. Using the stepwise method, the multiple regression model including BMI categories was reconstructed as follows: log_e_[CEA]=0.208+0.241[liver metastasis]+0.051 [differentiation]+0.092[TNM]; log_e_[CA19-9]=0.969+0.233 [gender]+0.141[ascites]+0.09[TNM]. The mean survival time in CEA^+^/CA19-9^−^, CEA^+^/CA19-9^+^, CEA^−^/CA19-9^−^ and CEA^−^/CA19-9^+^ patients was 84.8, 58.2, 100.6 and 74.7 months, respectively. The 1-/3-year survival rates in each group was 76.0/59.8, 66.2/43.5, 96.3/87.6 and 71.7/41.0, respectively. In conclusion, the decreased concentration of CEA and CA19-9 in patients of higher BMIs may be the result of the hemodilution effect. The BMI factor should be considered during the surveillance of colorectal cancer. In addition, patients with simultaneous positive expression of CEA and CA19-9 exhibited shorter survival time.

## Introduction

Carbohydrate antigen 19-9 (CA19-9) and carcinoembryonic antigen (CEA) have been well recognized as tumor markers for colorectal cancer (1). These markers are crucial in the routine clinical setting, including diagnosis, predicting prognosis and monitoring the effects of treatment. Numerous studies have demonstrated that colorectal cancer patients with elevated levels of CEA and CA19-9 have a significantly poorer prognosis compared with those with normal levels of these tumor markers (2,3). Serial CEA measurements may detect recurrent colorectal cancer with a sensitivity of 80% and a specificity of 70% and may provide a lead time of 5 months. CA19-9 has been reported to exhibit a sensitivity of 70–80% and a specificity of 80–90% (4). Elevated preoperative CEA values are associated with more advanced disease and worse outcome following surgical resection, regardless of the tumor stage and histological grade (5–7).

Despite the widespread use of monitoring serum CEA and CA19-9 levels during follow-up, their accuracy remains unclear. Certain non-malignant conditions, such as ageing, chronic renal failure, hypothyroidism, cigarette smoking, chronic obstructive pulmonary disease and obesity may be associated with alterations in serum CEA levels (8–12). The serum CA19-9 levels are also frequently elevated in patients with various gastrointestinal malignancies, such as pancreatic, colorectal, gastric and hepatic carcinomas. In addition, the serum CA19-9 levels may be elevated in certain non-malignant conditions (13).

According to previous studies, the serum concentration of soluble tumor markers in obese populations is lower compared with that in non-obese subjects (14,15). The larger vascular volume of obese individuals exerts a dilutional effect, a phenomenon known as hemodilution. However, the number of available studies investigating the association between CEA, CA19-9 and body mass index (BMI) is limited in China. Therefore, the aim of this study was to investigate the association of plasma volume with CEA and CA19-9 concentration in colorectal cancer patients.

## Materials and methods

### Patients

The collected records of 2,950 consecutive colorectal cancer patients between August, 1994 and December, 2005 were retrospectively reviewed. Analyses were confined to patients with BMI>16 kg/m^2^. The exclusion criteria were as follows: i) patients with unregistered data on BMI, CEA and CA19-9; ii) history of malignant disease or inflammatory bowel disease, renal insufficiency requiring hemodialysis or advanced stage of liver cirrhosis, cancer of mucinous or squamous histology, familial adenomatous polyposis, or synchronous colon cancer. The remaining 300 patients were included in the present analysis.

This study was approved by the Ethics Committee of Sun Yat-sen University (Guangzhou, China). Written informed consent was obtained from all patients.

### Clinical variables

Height and weight were objectively measured at admission and preoperative BMI was calculated as weight in kilograms divided by height in meters squared. In view of the differences in the recommended BMI cut-off points for overweight status and obesity between the Chinese and Western populations, the following categories were used: lower range of normal weight (BMI<18.5 kg/m^2^), normal weight (BMI=18.5–24.0 kg/m^2^) and overweight (BMI>24.0 kg/m^2^). The baseline serum CEA and CA19-9 concentrations were measured by enzyme immunoassay in a single laboratory at The Affiliated Hospital of Sun Yat-sen University. The estimated body surface area was calculated as follows: (body weight)^0.425^ × (height)^0.72^ × 0.007184. The estimated plasma volume (in liters) was calculated as body surface area × 1.670. The CEA and CA19-9 concentrations were measured in ng/ml. The CEA and CA19-9 mass (in micrograms), representing the total amount of CEA and CA19-9 protein within the circulation, was calculated as serum CEA and CA19-9 concentration × estimated plasma volume. The patients were followed up for at least 5 years or until death. The follow-up examinations included physical examination, serum carcinoembryonic antigen levels, chest X-rays, abdominal ultrasonography, or thoracoabdominal computed tomography performed at 6- or 12-month intervals.

### CEA and CA19-9 determination and patient scoring

Values of CEA ≥7 ng/ml were defined as abnormal and were scored as CEA^+^. Levels of CA19-9 ≥37 ng/ml were defined as abnormal and were scored as CA19-9^+^. Patients were divided into four groups according to the results of the two markers. The CEA/CA19-9 respective scores of the groups were CEA^+^/CA19-9^−^, CEA^+^/CA19-9^+^, CEA^−^/CA19-9^−^ and CEA^−^/CA19-9^+^. Survival was analyzed in terms of CEA and CA19-9.

### Statistical analysis

Pearson’s correlation coefficients were estimated to evaluate the association of serum CEA and CA19-9 levels with the clinical parameters. Due to the log-normal distribution, the serum CEA and CA19-9 levels were log-transformed for analysis. Correlation and regression analyses were performed to calculate the values and formulas to evaluate the association between clinical parameters and log-transformed serum levels. Multiple linear regression analyses were performed to assess whether clinical parameters significantly contributed to interpreting serum CEA and CA19-9 levels. Only the variables that were statistically significant (P<0.05) in the Pearson’s linear regression analysis were included in the multiple linear regression model. A stepwise method was used to select the explanatory variables based on analysis of variance.

The Kaplan-Meier method was used to calculate the cumulative survival rates and plot survival curves and the log-rank test was used to identify statistical differences between the curves. Survival time was calculated from the time of surgery to the last contact or death. To minimize the interpretation bias, overall survival analysis was performed. P<0.05 was considered to indicate a statistically significant difference.

## Results

### Patients and tumor characteristics

We investigated a total of 300 patients who underwent preoperative CEA and CA19-9 measurement and met our inclusion criteria: i) BMI>16 kg/m^2^, ii) no history of malignant disease, inflammatory bowel disease, renal insufficiency requiring hemodialysis, advanced stage of liver cirrhosis, cancer of mucinous or squamous histology, familial adenomatous polyposis, or synchronous colon cancer, and iii) complete clinical data. The mean age at surgery was 58.27 years. The mean BMI and preoperative CEA and CA19-9 concentrations were 21.20 kg/m^2^ (range, 13.65–32.87 kg/m^2^), 26.89 ng/ml (range, 0–1362 ng/ml) and 26.89 ng/ml (range, 0–17245.03 ng/ml), respectively. The demographic and clinicopathological characteristics of patients with different levels of CEA and CA19-9 are shown in Tables I and II. There was a statistically significant difference in the analysis of blood transfusion, peritoneal metastasis and TNM stage between the two groups. The groups were also compared for gender, age, tumor size, tumor location, liver metastasis and histological grade. In addition, the factors of ascites and TNM stage exhibited significant differences in the analysis of CA19-9.

### Cut-off values and prognostic significance of CEA and CA19-9

A higher BMI was shown to be significantly associated with higher plasma volumes (Table III). Compared with the normal-weight patients, the patients with BMI ≥24 had 10–15% higher plasma volumes. The association of BMI with CEA and CA19-9 mass was then investigated. The CEA and CA19-9 mass did not change significantly with increasing BMI, except for CEA in stage I (Table III). The proportion of patients with overall abnormal CEA and CA19-9 levels at each cut-off value was decreased with BMI (Table III). Moreover, there was no statistically significant difference in the proportion of patients with elevated CEA and CA19-9 levels by BMI category using different cut-off points. In the analysis for the recurrence cohort, patients with distant metastases (stage IV) were excluded. In addition, the sensitivity, specificity, positive predictive value (PPV) and negative predictive value (NPV) of preoperative CEA and CA19-9 measurements for tumor recurrence were calculated. The median follow-up period was 59.8 months (range, 1–122 months; Table IV). At a cut-off value of 2.5 ng/ml for preoperative CEA, the sensitivities of the lower range of normal weight (BMI<18.5 kg/m^2^), normal weight (BMI 18.5–24.0 kg/m^2^) and overweight were 33.9, 30.0 and 20.0, respectively (P=0.136). At serum concentrations >7.0 ng/ml, preoperative CEA concentrations were predicted with a sensitivity of 23.0%, specificity of 83.3%, PPV of 60.0% and NPV of 51.2% in the obese group. In addition, the specificity, PPV and NPV were not significantly different in the analysis of CA19-9 (Table V).

### Clinical interpretation of the effect of obesity on CEA and CA19-9 concentration

**Figure 1:**
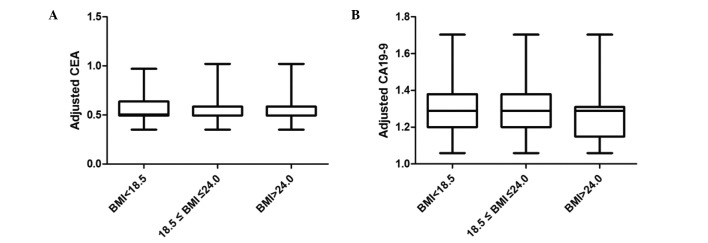
Adjusted CEA, CA19-9 results of the mathematical model. (A) Body mass index (BMI) <18.5, carcinoembryonic antigen (CEA)=0.55±0.13 ng/ml; 18.5≤BMI≤24.0, CEA=0.55±0.12 ng/ml; BMI≥24.0, CEA=0.54±0.12 ng/ml; P>0.05. (B) BMI<18.5, carbohydrate antigen 19-9 (CA19-9)=1.31±0.15 ng/ml; 18.5≤BMI≤24.0, CA19-9=1.28±0.12 ng/ml; BMI≥24.0, CA19-9=1.26±0.13 ng/ml; P<0.05. CEA, carcinoembryonic antigen; CA19-9, carbohydrate antigen 19-9.

Following determination of the association of BMI with CEA and CA19-9 concentration, we investigated the effect of overweight and obesity on this association in order to estimate the CEA and CA19-9 concentration in high-BMI patients corresponding to a CEA of 7.0 ng/ml in normal-weight patients. We reconstructed the multiple regression model including BMI categories. The following formula was obtained upon statistical analysis: log_e_[CEA]=0.208+0.241[liver metastasis]+0.051[differentiation]+0.092[TNM]; log_e_[CA19-9]=0.969+0.233[gender] +0.141[ascites]+0.09[TNM]. In this mathematical model, liver metastasis and ascites were granted points from 0 to 1 (i.e., liver metastasis positivity was granted 1 point); each differentiation was granted a point from 1 to 3 (i.e., high differentiation was granted 1 point and poor differentiation 3 points); each stage of colon cancer was granted a point from 1 to 4 (i.e., stage I was granted 1 point and stage IV 4 points). According to the results of the mathematical model, the adjusted CEA, CA19-9 is shown in Fig. 1.

### Comparison of survival time in different antigen patient groups

**Figure 2:**
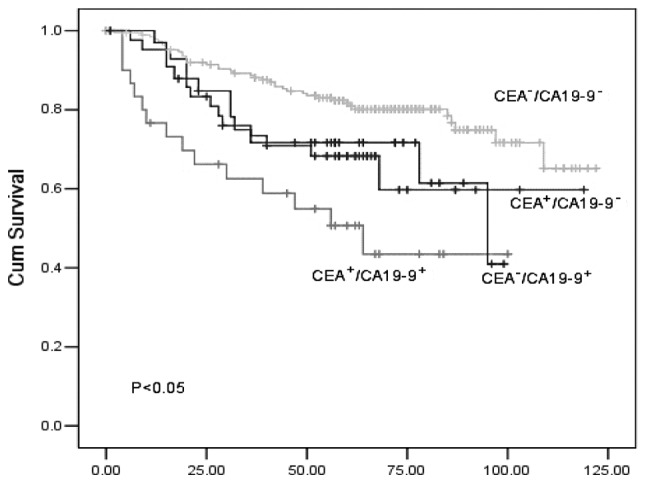
Disease-free survival curves stratified by preoperative serum carcinoembryonic antigen (CEA) and carbohydrate antigen 19-9 (CA19-9) concentrations. Cum, cumulative.

**Figure 3:**
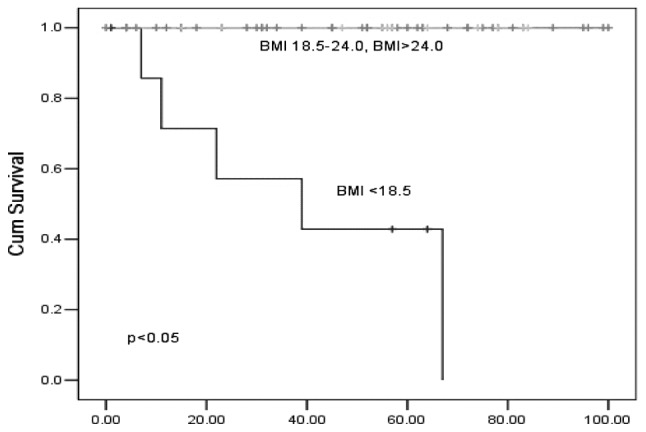
Disease-free interval following curative surgery for patients with colorectal cancer according to preoperative serum levels of carbohydrate antigen 19-9 (CA19-9) in different groups [body mass index (BMI) <18.5, 18.5–24.0 and >24.0]. Cum, cumulative.

A comparison of the survival curves of the four groups (CEA^+^/CA19-9^−^, CEA^+^/CA19-9^+^, CEA^−^/CA19-9^−^ and CEA^−^/CA19-9^+^) is presented in Fig. 2. The mean survival time in the four groups was 84.8, 58.2, 100.6 and 74.7 months, respectively. The 1-/3-year survival rates in each group were 76.0/59.8, 66.2/43.5, 96.3/87.6 and 71.7/41.0, respectively (Fig. 2). The survival of patients with BMI>18.5 who had high CA19-9 levels was significantly higher compared with that observed in patients with BMI<18.5 (Fig. 3). There was no statistical difference in the analysis of CEA and CA19-9 <37 ng/ml according to the classification of BMI (data not shown). In addition, a statistically significant difference was found in the analysis of CA19-9 ≥37 ng/ml.

## Discussion

The total amount of CEA and CA19-9 protein within the circulation was defined as CEA and CA19-9 mass. It was previously demonstrated that obese patients had higher plasma volumes, but not CEA or CA19-9 mass. This phenomenon is consistent with obesity-related hemodilution, stating that hemodilution may be affected by higher BMIs, which may decrease the serum concentrations of soluble tumor markers (16). Accordingly, our study demonstrated that the serum concentration of tumor markers in obese individuals was lower compared with normal-weight individuals. In our study, patients in the obese group (BMI>24.0 kg/m^2^) exhibited 20% lower serum CEA concentrations compared with normal-weight patients (18.5–24.0 kg/m^2^), which was consistent with the 5% decrease in the serum CEA concentration (14). Compared with our study, the previous investigation was not community-based and data were collected from patients undergoing routine heath screening, in which CEA values >5.0 ng/ml were excluded from the analysis.

The cut-off point is of great significance to the results and the cut-off value varies among different institutions. A lower optimum value was reported by a previous study that used 5 ng/ml as the cut-off point. In addition, a cut-off of 4.0 ng/ml may provide an appropriate balance of specificity and sensitivity according to the receiver operating characteristic curve analysis (17). In view of previous studies, it may be helpful to use multiple cut-off values to assess the effect of BMI on the interpretation of CEA and CA19-9 measurements. Our study suggests that the sensitivity of the CEA measurement was associated with BMI to a certain extent when the cut-off point was changed.

The analyses presented in this study demonstrate that patients with higher BMIs exhibited significantly lower screening CEA and CA19-9 levels. Moreover, it has been hypothesized that the levels of CEA and CA19-9 appear lower due to the dilution effect of the increased plasma volume associated with the increased BMI. Since the association between BMI and plasma volume was non-linear, we developed a model of obesity-related CEA and CA19-9 dilution, which accurately predicted the CEA and CA19-9 levels observed in our population, even after adjustment of the observed values for degree of differentiation and TNM stage. This model was used to estimate CEA values in overweight and obese patients. Our model revealed, through the comparison of the concentration of crude CA19-9 with that following adjustment, that the strength of the association between CA19-9 concentration and BMI was increased following adjustment. In addition, another finding was the lack of a significant association between obesity and CEA concentration in overweight and obese patients.

High preoperative serum CEA levels were associated with tumor recurrence. For patients with preoperative serum CEA levels >7.0 ng/ml and CA19-9 levels >37 ng/ml in the normal BMI group, the sensitivity, specificity, PPV and NPV for tumor recurrence were lower compared with those reported in a previous study (18). In addition, compared with the obese group, the sensitivity, specificity and PPV of preoperative CEA and CA19-9 levels at each cut-off point was reduced. The observed absolute differences were not modest and may be attributed to the number of patients enrolled in our study. The preoperative serum level of CA19-9 was a better predictor for recurrence compared to CEA according to our study, which was similar to previously reported findings (19). The serum levels of CEA and CA19-9 were associated with low survival rates. Our study demonstrated that there was a significant difference in the 10-year patient survival curves between serum CEA and CA19-9 levels. Furthermore, significant differences were observed in serum CEA^+^/CA19-9^+^ and CEA^−^/CA19-9^−^ patients, which was consistent with previous findings (20).

A high BMI is a well-established prognostic factor of poor survival. However, the results obtained from our study demonstrated that the prognosis of patients with BMI<18.5 was worse compared with the other groups. This discrepancy may be explained as follows: i) increased mortality due to low BMI was associated with the consequences of poor underlying nutritional status; ii) the number of patients in need of blood transfusions in the low BMI group was higher than that of the other groups. In addition, blood transfusion has been described as exerting a negative effect after hepatectomy for colorectal cancer (21).

The findings of our study suggest that colorectal cancer may be less likely to be detected in obese individuals, partly due to the effect of BMI on the hemodilution of CEA and CA19-9. Therefore, the weight of the patients should be considered when interpreting CEA and CA19-9 screening results. To minimize this detection bias, it may be prudent to lower cut-offs for overweight or obese patients. There were also certain limitations to our study. First, weight and height were used to calculate plasma volume, which may be replaced by more accurate methods, such as algorithms using lean body mass and hematocrit used for plasmapheresis (22). The hemodilution due to the increased plasma volume may be associated with the decreased serum CEA and CA19-9 concentration observed in patients of higher BMIs. Second, our patient sample was somewhat limited. Third, the association between BMI and sensitivity of CEA and CA19-9 were not analyzed following surgery, since data on individual weight alterations were not included.

Further studies are required to determine whether these screening cut-off points exhibit similar sensitivity and specificity for predicting cancer in patients of varying body size. Since early detection of colorectal cancer is the goal of CEA and CA19-9 measurement, sensitivity and specificity testing are required prior to adopting new screening cut-off points.

In conclusion, this study indicates that the obesity epidemic may have negatively affected the efficacy of colorectal cancer screening methods and provides a theoretical framework for elucidating the effect of BMI variations.

## Figures and Tables

**Table I:** Analysis of demographic and clinicopathological characteristics according to serum carcinoembryonic antigen (CEA) levels in patients with colorectal cancer.

Characteristics	CEA		P-value for trend

	<7 ng/ml	≥7 ng/ml	
Gender			0.438
Female	94 (41.4)	34 (46.6)	
Male	133 (58.6)	39 (53.4)	
Age (years)			0.881
<60	119 (52.4)	39 (53.4)	
≥60	108 (47.6)	34 (46.6)	
Radical operation			0.223
No	33 (14.5)	15 (20.5)	
Yes	194 (85.5)	58 (79.5)	
Blood transfusion			0.029
No	178 (78.4)	48 (65.8)	
Yes	49 (21.6)	25 (34.2)	
Histological type			0.249
Villous adenocarcinoma	17 (7.5)	5 (6.8)	
Tubular adenocarcinoma	172 (75.8)	60 (82.2)	
Mucinous adenocarcinoma	20 (8.8)	7 (9.6)	
Other	18 (7.9)	1 (1.4)	
Ascites			0.066
No	207 (91.2)	61 (83.6)	
Yes	20 (8.8)	12 (16.4)	
Tumor size (cm)			0.053
≤5	144 (63.4)	37 (50.7)	
>5	83 (36.6)	36 (49.3)	
Tumor location			0.185
Colon	98 (43.2)	38 (52.1)	
Rectum	129 (56.8)	35 (47.9)	
Peritoneal metastasis			0.011
No	224 (98.7)	68 (93.2)	
Yes	3 (1.3)	5 (6.8)	
Liver metastasis			0.10
No	216 (95.2)	63 (86.3)	
Yes	11 (4.8)	10 (13.7)	
TNM stage			0.000
I	40 (17.6)	4 (5.5)	
II	100 (44.1)	25 (34.2)	
III	76 (33.5)	30 (41.1)	
IV	11 (4.8)	14 (19.2)	
Histological differentiation			0.085
High	19 (8.4)	4 (5.5)	
Moderate	188 (82.8)	56 (76.7)	
Poor	20 (8.8)	13 (17.8)	

Data in parentheses represent percentage values.

**Table II:** Analysis of demographic and clinicopathological characteristics according to serum carbohydrate antigen 19-9 (CA19-9) levels in patients with colorectal cancer.

Characteristics	CA19-9		P-value for trend

	<37 ng/ml	≥37 ng/ml	
Gender			0.430
Female	103 (43.8)	25 (38.5)	
Male	132 (56.2)	40 (61.5)	
Age (years)			0.830
<60	112 (47.7)	30 (46.2)	
≥60	123 (52.3)	35 (53.8)	
Blood transfusion			0.509
No	175 (74.5)	51 (78.5)	
Yes	60 (25.5)	14 (21.5)	
Ascites			0.021
No	215 (91.5)	53 (81.5)	
Yes	20 (8.5)	12 (18.5)	
Tumor size (cm)			0.357
≤5	145 (61.7)	36 (55.4)	
>5	90 (38.3)	29 (44.6)	
Tumor location			0.119
Colon	101 (43)	35 (53.8)	
Rectum	134 (57)	30 (46.2)	
Peritoneal metastasis			0.817
No	229 (97.4)	63 (96.9)	
Yes	6 (2.6)	2 (3.1)	
Liver metastasis			0.178
No	221 (94)	58 (89.2)	
Yes	14 (6)	7 (10.8)	
TNM stage			0.007
I	42 (17.9)	2 (3.1)	
II	100 (42.6)	25 (38.5)	
III	76 (32.3)	30 (46.2)	
IV	17 (7.2)	8 (12.3)	
Histological differentiation			0.290
High	20 (8.5)	3 (4.6)	
Moderate	192 (81.7)	52 (80)	
Poor	23 (9.8)	10 (15.4)	

Data in parentheses represent percentage values.

**Table III:** Plasma volume and carcinoembryonic antigen (CEA) and carbohydrate antigen 19-9 (CA 19-9) mass according to body mass index (BMI) category.

Stage	BMI category (kg/m^2^)			P-value for trend

	<18.5	18.5≤BMI≤24.0	>24	
Plasma volume, liters (SD)				
I	2.43 (0.15)	2.64 (0.23)	2.78 (0.25)	0.002
II	2.30 (0.26)	2.59 (0.21)	2.87 (0.21)	0.000
III	2.41 (0.19)	2.52 (0.19)	2.78 (0.25)	0.000
IV	2.45 (0.13)	2.60 (0.18)	2.83 (0.32)	0.000
CEA mass, μg (IQR)				
I	4.2 (3.17–5.15)	6.42 (4.60–10.69)	6.07 (3.37–12.25)	0.049
II	8.09 (4.96–13.00)	7.31 (4.70–14.84)	12.80 (5.00–21.24)	0.513
III	13.13 (4.62–26.30)	9.60 (3.98–19.31)	6.56 (3.61–19.80)	0.095
IV	21.58 (6.65–49.96)	30.41 (9.12–76.14)	35.27 (10.64–98.27)	0.514
CA19-9 mass, μg (IQR)				
I	36.52 (26.96–57.34)	42.69 (27.92–59.98)	50.71 (16.06–80.35)	0.722
II	60.45 (26.74–69.86)	51.26 (29.21–79.42)	69.55 (37.49–103.71)	0.346
III	57.56 (34.06–112.95)	60.72 (22.30–103.04)	52.13 (30.72–125.08)	0.243
IV	74.91 (62.13–76.68)	71.06 (33.17–362.25)	78.94 (64.05–101.18)	0.528

Data are presented as mean (SD) or median (IQR), according to normal distribution. SD, standard deviation; IQR, interquartile range.

**Table IV:** Proportion of patients with elevated carcinoembryonic antigen (CEA) and carbohydrate antigen 19-9 (CA19-9) concentration by three different cut-off points according to body mass index (BMI).

Cut-off values (ng/ml)	BMI category (kg/m^2^)			P-value for trend

	<18.5	18.5≤BMI≤24.0	>24	
CEA cut-off value (%)				
>2.5	56 (93.3)	170 (89.4)	45 (90.0)	0.525
>5.0	40 (66.7)	138 (72.6)	37 (74.0)	0.716
>7.0	30 (50.0)	96 (50.5)	26 (52.0)	0.191
CA19-9 cut-off value (%)				
>13	52 (86.6)	158 (83.1)	45 (90.0)	0.688
>26	48 (80.0)	145 (76.3)	43 (86.0)	0.504
>37	41 (68.3)	124 (65.2)	38 (76.0)	0.442

Stratified according to the WHO recommendations for the Chinese population for international comparison.

**Table V:** Sensitivity, specificity, positive predictive value and negative predictive value of high preoperative serum carcinoembryonic antigen (CEA) and carbohydrate antigen 19-9 (CA19-9) for recurrence.

Cut-off value (ng/ml)	BMI category (kg/m^2^)			P-value for trend

	<18.5	18.5≤BMI≤24.0	>24	
CEA low >2.5 (%)				
Sensitivity	19/56 (33.9)	51/170 (30.0)	9/45 (20.0)	0.136
Specificity	3/4 (75.0)	16/20 (80.0)	4/5 (80.0)	0.865
PPV	19/20 (95.0)	51/55 (92.7)	9/10 (90.0)	0.608
NPV	3/40 (7.5)	16/135 (11.8)	4/39 (10.2)	0.688
CEA high >7.0 (%)				
Sensitivity	10/30 (33.3)	37/96 (38.5)	6/26 (23.0)	0.465
Specificity	20/30 (66.7)	76/94 (80.8)	20/24 (83.3)	0.119
PPV	10/20 (50.0)	31/55 (56.3)	6/10 (60.0)	0.567
NPV	20/40 (50.0)	76/135 (56.2)	20/39 (51.2)	0.903
CA 19-9 low >13 (%)				
Sensitivity	16/52 (30.7)	45/158 (28.4)	9/45 (20.0)	0.247
Specificity	4/8 (50.0)	22/32 (68.7)	4/5 (80.0)	0.236
PPV	16/20 (80.0)	45/55 (81.8)	9/10 (90.0)	0.546
NPV	4/40 (10.0)	22/135 (16.2)	4/39 (10.2)	0.964
CA 19-9 low >37 (%)				
Sensitivity	12/41 (29.2)	39/124 (31.4)	8/38 (21.0)	0.438
Specificity	11/19 (57.8)	50/66 (75.7)	10/12 (83.3)	0.092
PPV	12/20 (60.0)	39/55 (70.9)	8/10 (80.0)	0.235
NPV	11/40 (27.5)	50/135 (37.0)	10/39 (25.6)	0.873

BMI, body mass index; PPV, positive predictive value; NPV, negative predictive value.
